# Comparison of an interactive voice response system and smartphone application in the identification of gout flares

**DOI:** 10.1186/s13075-019-1944-5

**Published:** 2019-06-29

**Authors:** Nada Elmagboul, Brian W. Coburn, Jeffrey Foster, Amy Mudano, Joshua Melnick, Debra Bergman, Shuo Yang, David Redden, Lang Chen, Cooper Filby, Jeffrey R. Curtis, Ted R. Mikuls, Kenneth G. Saag

**Affiliations:** 1 University of Alabama at Birmingham, 1720 2nd Avenue South, Birmingham, AL 35294 USA; 2 University of Nebraska Medical Center and VA Nebraska-Western Iowa Health Care System, Omaha, NE USA

**Keywords:** Interactive voice response system, Smartphone application, Gout flares

## Abstract

**Objective:**

To examine the feasibility, preference, and satisfaction of an interactive voice response (IVR) system versus a customized smartphone application (StudyBuddy) to capture gout flares

**Methods:**

In this 24-week prospective, randomized, crossover, open-label pilot study, 44 gout patients were randomized to IVR vs. StudyBuddy and were crossed over to the other technology after 12 weeks. Flares were reported via weekly (and later daily) scheduled StudyBuddy or IVR queries. Feasibility was ascertained via response rate to scheduled queries. At 12 and 24 weeks, participants completed preference/satisfaction surveys. Preference and satisfaction were assessed using dichotomous or ordinal questions. Sensitivity was assessed by the frequency of flare reporting with each approach.

**Results:**

Thirty-eight of 44 participants completed the study. Among completers, feasibility was similar for IVR (81%) and StudyBuddy (80%). Conversely, most (74%) preferred StudyBuddy. Measures of satisfaction (ease of use, preference over in-person clinic visits, and willingness for future use) were similar between the IVR and StudyBuddy; however, more participants deemed the StudyBuddy as convenient (95% vs. 73%, *P* = 0.01) and less disruptive (97% vs. 82%, *P* = 0.03). Although the per patient number of weeks in flare was not significantly different (mean 3.4 vs. 2.6 weeks/patient, *P* = 0.15), the StudyBuddy captured more of the total flare weeks (35%) than IVR (27%, *P* = 0.02).

**Conclusion:**

A smartphone application and IVR demonstrated similar feasibility but overall sensitivity to capture gout flares and participant preference were greater for the smartphone application. Participant preference for the smartphone application appeared to relate to perceptions of greater convenience and lower disruption.

**Trial registration:**

NCT, NCT02855437. Registered 4 August 2016

## Background

Flare prevention represents a major tenet of effective gout management. Gout flares are the key patient-reported outcome measure in randomized controlled clinical trials (RCTs) in gout [1–5]. However, multiple RCTs of urate-lowering therapies (ULT) have failed to detect differences in gout flares between treatment arms [6, 7]. This evidence gap around a clear flare reduction in clinical trials is one of the reasons [8] that the 2016 American College of Physicians (ACP) [9] clinical guidelines for gout management failed to endorse the “treat-to-target” approach (lowering and maintaining serum urate concentrations below 5 to 6 mg/dl) recommended by the American College of Rheumatology (ACR) [10] and the European League Against Rheumatism (EULAR) [11].

However, capturing gout flares in RCTs poses a substantial methodologic challenge, especially since these events usually occur between, rather than during, study visits. An additional limitation of using gout flares as an outcome in RCTs is the absence of a standardized method of flare ascertainment. Current methods used to capture gout flares, such as the use of patient diaries [12], patient self-report [1], or retrospectively during scheduled physician visits [6], have many practical limitations given their resource intensive nature and the limited veracity of the resultant data collected.

With the dramatic increased use of cell phones and other portable devices, mobile technology is more frequently being leveraged in healthcare management and has increasingly been deployed for remote data collection in the clinical trial and practice settings [13]. For example, smartphone applications have been used to track data and monitor some diseases like rheumatoid arthritis, psoriatic arthritis, and diabetes [14, 15] or symptoms such as pain [16, 17]. It demonstrated success in patient engagement and data collection. An alternative and less technologically intensive method to capture patient-reported outcomes remotely is an automated phone survey using an interactive voice response (IVR) system [18]. The automated IVR telephone system delivers prerecorded surveys that participants answer through verbal or touch screen response. IVR surveys have been used for clinical studies, disease management, medication adherence monitoring, and health education [19, 20].

Given concerns about the absence of reliable gout flare capture in RCTs, the purpose of our project was to develop and test two novel methods to promptly and efficiently detect gout flares for use in future clinical trials. In this study, we compared IVR and a custom smartphone application (StudyBuddy) for capturing gout flares. We examined the feasibility, patient preference, satisfaction, and sensitivity for detecting flare using the two methods.

## Methods

### Study population and design

**Fig. 1:**
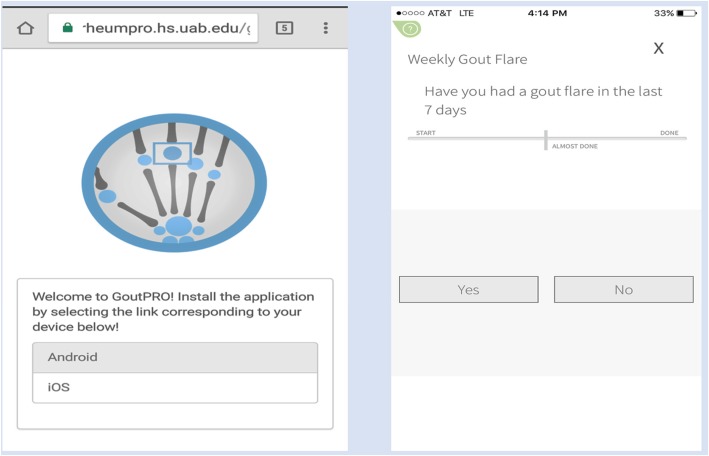
Screenshots of the StudyBuddy (GoutPRO) application

We conducted a 24-week, prospective, randomized, crossover, open-label pilot study comparing participants’ preference and feasibility of using interactive voice response (IVR) telephone questionnaire/flare reporting system versus a mobile smartphone application developed by investigators at the University of Alabama at Birmingham, GoutPRO (Birmingham, AL), now known as StudyBuddy, to capture patient-reported outcomes and data (Fig. 1). The trial was approved by the Institutional Review Boards at the two academic medical centers conducting this study. Patients were enrolled at UAB and at the University of Nebraska Medical Center (UNMC, Omaha, NE) rheumatology clinics from September of 2016 to March of 2018. Patient eligibility criteria included age 18 years and older (19 years or older in Nebraska), physician-diagnosed gout, and hyperuricemia with serum urate level ≥ 6.8 mg/dl within the 3 months of screening in the absence of interval changes in ULT dosing. Other inclusion criteria included the self-report of > 2 flares in the previous 6 months and current use of an Apple™ or Android™ smartphone with the ability to download the StudyBuddy smartphone application.

### Interventions and data collection

Eligible participants providing consent were randomized to either IVR followed by StudyBuddy or StudyBuddy followed by IVR using a permutated block randomization to achieve balance between crossover arms in a 1:1 allocation. At the initial visit, depending on randomization assignment, participants installed and tested the StudyBuddy application on their smartphones, or received an IVR call.

During each12-week intervention period, participants completed the same gout flare survey questions via weekly scheduled StudyBuddy interactions or programmed IVR calls at the time of their choice. A subgroup of 15 participants in the IVR arm was randomly assigned to receive daily programmed IVR calls. The weekly IVR calls were to be repeated within 1, 2, 24, and 48 h of the original call (in cases of no response) while the daily IVR calls were repeated in 1 and 2 h as necessary. Flare assessment was based on validated gout flare survey questions consistent with the published gout flare self-report definition of Gaffo and colleagues [21]. This definition has 85% sensitivity and 95% specificity in confirming the presence of a gout flare. It requires fulfillment of at least 3 of 4 criteria (patient-defined gout flare, pain at rest score of > 3 on a 0–10-point numerical rating scale, presence of ≥ 1 swollen joint, and presence of ≥ 1 warm joint). Our survey questions included (1) whether the recent flare was similar to past flares, (2) the number of swollen/warm joints, (3) pain at rest during the flare (assessed using a 0–10 numerical rating scale), and (4) additional questions specific to each flare including peak pain levels, location of pain (i.e., which joints), timing of the flare, and its duration. After the first 12 weeks (phase 1), study participants were crossed over to receive the alternative mode of gout flare assessment and continued in the study for an additional 12 weeks (phase 2). A washout period between methods was not utilized since no protracted risk of behavioral change was anticipated. At 12 weeks (crossover) and 24 weeks (end of study), participants completed satisfaction surveys, as well as a questionnaire about their preferred mode of reporting gout flares.

### Outcomes

#### Primary outcomes

We compared IVR and the customized StudyBuddy smartphone application in this pilot study using three primary domains of interest: (1) feasibility, (2) preference, and (3) satisfaction. Feasibility was ascertained via proportion of responses to the scheduled queries with each approach during the study period. Preference was calculated as the proportion of total study population preferring IVR or StudyBuddy. Satisfaction was calculated using six separate self-reported measures extracted from the customizable Health Information Technology (IT) Usability Evaluation Scale (Health-ITUES) at 12 and 24 weeks. Measures of satisfaction included the participant ratings of (1) ease of use, (2) disruption to daily activities, (3) ability to respond to alerts (or convenience of alerts), (4) preference of either device to a clinic visit, (5) satisfaction with the contact frequency, and (6) willingness for future use (acceptability) [22]. The feasibility and satisfaction measures were also tested in the subgroup with daily IVR calls to test for differences compared to the weekly group.

#### Secondary outcomes

We also considered the relative frequency of gout flare reporting as a component of the sensitivity of each method to capture relevant flare events. In doing so, we made the assumption that since we randomized patients to the sequence of 12-week time periods (i.e., app vs. IVR for 12 weeks, then crossover), the frequency of flares should be similar in both time periods, and thus any difference in the reported flare rates was a consequence of greater sensitivity of the technology to capture flare. We compared the frequency of flare reporting with each approach. We assessed flares using the aforementioned questionnaire and reported as the number of weekly sampling periods per participant characterized by flare in addition to the total proportion of weeks for all individuals characterized by a flare. In addition, at the end of the 24-week study period, an open-ended qualitative questionnaire about common causes for not responding to alerts was collected from participants via a structured telephone interview with questions focused on smartphone application issues, connection issues, and time convenience.

### Statistical analysis

We summarized baseline characteristics of study participants as means with standard deviation (SD) for continuous and ordinal measures and number and percentage for categorical variables. All statistical analyses followed standard methods for a 2-by-2 crossover design [23]. Preference and satisfaction surveys used dichotomous questions, except the ease-of-use question which utilized a Likert scale (range 0 to 10; very easy to very difficult) and was analyzed as an ordinal variable. Differences between the StudyBuddy and IVR approach were evaluated using McNemar’s test for dichotomous variables, paired *t* test for continuous variables, and nonparametric Wilcoxon signed rank test for ordinal variables. Qualitative questionnaire data for lack of response were summarized as percentages. Carryover effects were examined using the standard two-sample *t* test.

We conducted sensitivity analyses with multiple imputations to examine the robustness of the reported results and the effects of missing data for both the as-treated (*n* = 38) and intent-to-treat populations (*n* = 44). We considered missing data to be missing at random. We also performed a complete case analysis with case-wise deletion. A two-sided alpha of 0.05 was used to determine significance. Since the study was a pilot study to demonstrate feasibility of recruiting and training gout patients to utilize StudyBuddy and IVR, a priori sample size calculations were not performed. All analyses were performed with SAS (version 9.4, SAS, Cary, NC).

## Results

Of 48 participants screened, 44 were enrolled, 1 had no smartphone, 1 had < 2 flares within 6 months, and 2 decided against participation. By the end of the study, 38 completed both study arms, 1 withdrew immediately post-randomization, 3 (1 IVR, 2 StudyBuddy) were lost to follow-up during phase 1 [baseline to week 12], and 2 (1 IVR, 1 StudyBuddy) were lost to follow-up during phase 2 [week 13 through week 24]. The lost to follow-up participants did not complete the surveys and were unresponsive to follow-up calls. There were no significant differences observed in age, sex, or race between the 38 completers and the 6 individuals failing to complete the study. Participants were predominantly middle-aged men. Nearly half of the participants reported 2–3 gout flares within 6 months prior to enrollment with the remainder reporting four or more flares and nearly all were on ULT at enrollment (Table 1).

**Table 1:** Baseline characteristics of participants completing the study (*N* = 44)

Age, years, mean (SD)	49.3 (14.5)
Male sex, *N* (%)	37 (84%)
White race*	30 (68%)
Black or other race*	14 (32%)
Education level, *N* (%)	
Less than high school	1 (2%)
High school or more	43 (98%)
Age at first gout flare (years)^†^, mean (SD)	38.3 (18.6)
Duration of gout (years)^†^, mean (SD)	10.5 (7.9)
Number of flares in prior 6 months, *N* (%)	
2–3	19 (43%)
≥ 4	25 (57%)
Gout medication use, *N* (%)*	
Urate-lowering therapy	37 (84%)
NSAID or colchicine	28 (64%)
Prednisone	13 (30%)
Number of smartphone applications on cellphone^†^, mean (SD)	21.2 (16.1)

Urate-lowering therapy included allopurinol, febuxostat, or probenecid, and this use was not mutually exclusive to other gout medications

*NSAID* non-steroidal anti-inflammatory

*Two participants declared Hispanic ethnicity

^†^Missing data *n* = 2

### Primary outcomes

The two methods of flare capture demonstrated similar feasibility with participants responding to 81% of IVR queries and 80% of StudyBuddy queries (*P* = 0.94) (Table 2). At study completion, 28 (74%) preferred StudyBuddy delivered via smartphone, only 3 (8%) preferred IVR, and 7 (18%) had no preference. In assessments of satisfaction, the StudyBuddy was rated by participants as being numerically superior to IVR in terms of ease of use (0.58 ± 1.16 vs. 1.63 ± 2.5) [where 0 is very easy to use], although this difference did not achieve statistical significance (*P* = 0.79). Participants also reported the StudyBuddy to be more convenient, with 95% of participants reporting being able to answer questions when alerted, compared to 73% with IVR (*P* = 0.01). Moreover, participants found StudyBuddy to be disruptive < 3% of the time compared to 18% with IVR (*P* = 0.03). More than 80% of the participants preferred using either of the remote devices for reporting gout flare over in-person physician appointments. There was also no difference in the willingness for future use in both arms (Table 3). When we imputed missing data for measures of feasibility and satisfaction in both the as-treated and intent-to-treat populations, we found no significant difference in results. The complete case analysis with case-wise deletion also led to no changes in our overall results (data not shown).

**Table 2:** Feasibility and flare (sensitivity) data in IVR and StudyBuddy

	IVR (*N* = 38)	StudyBuddy (*N* = 38)	*P* value
Feasibility (weekly response to interactions or calls), mean % (± SD)	81% (21%)	80% (25%)	0.94
Gout flares			
Weeks with a flare (per patient, mean (± SD)	2.6 (± 2.5)	3.4 (± 3.3)	0.15
Total flare weeks (% of study time with flares)	27%	35%	0.02

*IVR* interactive voice response system, *StudyBuddy* smartphone mobile application

**Table 3:** Satisfaction measures comparing IVR and StudyBuddy

Technology satisfaction measures	IVR	StudyBuddy	*P* value
Ease of use (0–10; very easy to very difficult), mean (± SD)	1.63 (± 2.5)	0.58 (± 1.2)	0.13
Did not disrupt activity (i.e., nonintrusive), *N* (%)	31 (81.6%)	37 (97.4%)	0.03
^†^Convenience of alerts, *N* (%)	27 (72.9%)	36 (94.7%)	0.01
^†^Preference of reporting via device compared to clinic visit, *N* (%)	32 (86.5%)	35 (92.1%)	0.32
Satisfaction with frequency of contact, *N* (%)	34 (89.5%)	34 (89.5%)	1.00
^†^Willing to use in future, *N* (%)	34 (89.5%)	36 (97.3%)	0.16

*IVR* interactive voice response system, *StudyBuddy* smartphone mobile application

^†^Missing data *n* = 1

Data on more frequent daily ascertainment involving more than a third of the participants in the IVR arm had little impact on measures of satisfaction, with the exception that participants were more likely to respond to weekly (87.0%) alerts compared to daily alerts (70.2%) (*P* < 0.05).

### Secondary outcomes

The proportion of total weeks of observation with flares reported differed significantly per arm, with StudyBuddy weeks capturing more flares (35%) compared to IVR weeks (27%; *P* = 0.02). Likewise, the mean (± SD) number of weeks per participant characterized by a gout flare was numerically higher with StudyBuddy than IVR (3.4 ± 3.3 vs. 2.6 ± 2.5), although this difference did not achieve statistical significance (*P* = 0.15) (Table 2).

The reasons offered for not responding to the surveys in a timely fashion were mostly secondary to “inconvenience” associated with the timing of an alert, reported in 15 of 37 responses (40.5%). Other reasons noted included application malfunction (10.8%), smartphone running out of data/phone dysfunction (10.8%), or poor internet connection or reception. No carryover effects during phase 2 were identified.

## Discussion

We found that both an IVR and customized smartphone application (StudyBuddy) were equally feasible when used in reporting gout flares. The StudyBuddy smartphone application was preferred by more patients and appeared to capture more total flares than the IVR. Participants also found the StudyBuddy smartphone application to be more convenient than IVR. Over 80% of participants in both arms reported that neither flare-reporting technology was disruptive. However, a small but significantly greater number of participants found the smartphone application less disruptive than IVR. Overall, both methods were reported to be easy to use and both were strongly preferred over an in-person clinic visit for gout flare reporting, and rendered similar willingness for future use in studies.

The feasibility of our smartphone application and IVR was generally consistent with that reported in prior studies that leveraged these technologies in pain monitoring, deploying nutritional assessment tools and medication adherence [24–26]. For instance, a prior study of patients with sickle cell disease reported a 75% compliance rate with daily queries with the use of a customized smartphone application [24]. Similarly, a feasibility study of a smartphone application for sleep apnea patients [27] demonstrated good feasibility with > 60% of participants completing the daily questionnaire, > 65% of the study time, and with a 94% satisfaction rates. Another small 3-month feasibility study in rheumatoid arthritis [28] measured the self-assessment of daily disease activity using a smartphone application. The vast majority of these participants (~ 88%) had no issues reporting their daily symptoms, and all were confident sharing their information. Nevertheless, challenges with reporting adherence and attrition have also been noted with smartphone applications [29, 30], a phenomenon that we saw in our study and would be anticipated in longer studies. In comparison with smartphone applications, the feasibility of IVR for collecting daily queries of patient-reported outcomes has been reported in different diseases. In a randomized clinical trial of a pain medication, 85% of the daily surveys were successfully completed by participants [31]. Another feasibility study on prostate cancer survivors demonstrated 87% response to IVR queries regarding the quality of life [32].

IVR is a well-tested approach to communicate instructions, collect health information, send reminders, and complete surveys to improve healthcare services [19]. Despite being slightly less favorably received, when we compared it to StudyBuddy in our study, it was still judged as being similarly acceptable for future use and far more preferred than an in-person clinic visit. This parallels findings from previous studies examining different patient populations where IVR use was also found to be preferable [33, 34] and acceptable for future use [35, 36]. The fact that > 80% of our participants reported the IVR was not disruptive and > 70% reported it as convenient may have been associated with pre-scheduled call times established according to the participants’ preference. To our knowledge, IVR has not previously been used to capture patient-reported outcomes, including gout flare, in arthritis studies. This technology may be of particular benefit among gout patients lacking access to or less comfortable with the use of smartphones or other more advanced technologies. The IVR approach has other advantages including ease of use, real-time data collection, cost effectiveness, and independence of patient literacy. However, it still requires adequate telephone access.

The higher participant preference for the StudyBuddy was consistent with higher ratings received in relation to its perceived convenience and lower disturbance rate. Similar to other studies in pain [37] and sickle cell disease [24] that utilized a similar approach, our study also showed that patients found the smartphone application to be acceptable with excellent response rates, underscoring its potential feasibility in clinical trials as well as in day-to-day practice. Similar findings were also seen in a juvenile idiopathic arthritis study, where a different smartphone application was used to remotely promote for self-management with good acceptability by pediatric patients and parents alike [38]. The smartphone application shares similar properties to the IVR. However, it provides more flexibility in collecting data, with the potential for real-time feedback. The smartphone technology also offers the ability to assess certain physiological parameters such as step counts and sleep. As a drawback noted above, a smartphone application use requires more fluency with computer technology. Other challenges of the smartphone technology include privacy concerns with data transmission [39]. The younger age of our cohort might partially explain the slight preference for the StudyBuddy. As a further general limitation, both the smartphone and the IVR lack a direct person-to-person communication [40]. Although smartphone applications have been previously developed to promote self-efficacy in gout [13], we are aware of no other study that systemically examined this technology as a means of remotely capturing gout flare in patients.

The difference observed in the total proportion of weekly queries characterized by flare suggests that the customized smartphone application used in our study yielded a higher sensitivity for flare capture than IVR. Whether this difference relates to the greater convenience and flexibility in responding to alerts with this application is unknown, although our results suggest this to be one possible explanation. This trend was also observed in per patient flare reporting, where use of the StudyBuddy also yielded more flare reports, even though this difference did not achieve significance likely owing to the limited sample size and high variability observed with these events. Although our results suggest that the StudyBuddy provides a more sensitive means of flare capture than IVR, larger studies will be needed to validate this finding.

To our knowledge, this is among the first studies to use smartphones to capture self-reported gout flares. Despite its strengths, particularly in terms of crossover design and innovation, there are limitations to the study. Designed and implemented as a pilot proof-of-concept study, the sample size was small and this limited power to detect differences for some of the outcomes examined, as expected in the design phase. During the conduct of this study, we encountered technical issues with approximately 1 in every 5 participants reporting at least sporadic issues related to poor internet network connectivity or application malfunction. Though infrequent and highly informative for future study planning, these technical issues could serve as a source of study response bias.

## Conclusion

In summary, different technologies may offer variable benefits for gout flare assessment in future clinical trials since both forms of technology were equally feasible and reasonably well accepted. Our smartphone application, StudyBuddy, appeared to provide greater sensitivity, and our study participants found it to be more convenient and “user friendly” for gout flare reporting. While our pilot demonstrated the utility of using these technology tools to record self-reported gout outcomes, future studies examining patient preferences and satisfaction will be required in different populations to demonstrate both generalizability and reproducibility of our results as well as to better define the role of such technologies in future gout management trials.

## Data Availability

The datasets generated and/or analyzed during the current study are not publicly available due to complexity of data coding and data transformations needed for analyses but are available from the corresponding author on reasonable request.

## Abbreviations

ACP: American College of Physicians
ACR: American College of Rheumatology
EULAR: European League Against Rheumatism
IVR: Interactive voice response
NSAID: Non-steroidal anti-inflammatory
RCTs: Randomized controlled trials
StudyBuddy: Smartphone mobile application
ULT: Urate-lowering therapies
